# Staged surgical management of an acute angle-closure crisis with medically refractory intraocular pressure elevation: A case report and literature review

**DOI:** 10.1097/MD.0000000000051326

**Published:** 2026-10-30

**Authors:** Yuecong Yin, Yingjie Chen, Xiaofeng Zhang, Yu-Feng Yao

**Affiliations:** a Department of Ophthalmology, The Fourth Affiliated Hospital of Soochow University, Suzhou, China.

**Keywords:** acute angle-closure crisis, acute primary angle closure, intraocular pressure, lens extraction, surgical peripheral iridectomy

## Abstract

**Rationale:**

Acute angle closure with markedly elevated intraocular pressure (IOP) is an ocular emergency. Initial management aims to lower IOP, reduce corneal edema and inflammation, and permit definitive relief of pupillary block, usually by laser peripheral iridotomy (LPI). When IOP remains uncontrolled and corneal edema or an extremely shallow anterior chamber precludes safe LPI, urgent surgical intervention may be required.

**Patient concerns:**

A 61-year-old woman presented with a 7-hour history of pain, redness, and blurred vision in the right eye (RE), accompanied by headache.

**Diagnoses:**

Acute primary angle closure in the RE and primary angle-closure suspect status in the left eye (LE).

**Interventions:**

During initial treatment with topical pilocarpine, carteolol, and brinzolamide, IOP in the RE increased from 32 to 58 mm Hg. It remained markedly elevated despite 2 intravenous infusions of 20% mannitol (50 grams each), and emergency surgical peripheral iridectomy was performed. After the acute episode stabilized, the RE underwent phacoemulsification with intraocular lens implantation; the fellow eye subsequently underwent lens extraction with intraocular lens implantation.

**Outcomes:**

After peripheral iridectomy, IOP in the RE decreased from 58 to 11 mm Hg without additional IOP-lowering medication, and central anterior chamber depth increased from 1.24 to 1.60 mm. At 6 months after subsequent bilateral lens extraction, IOP was 14 mm Hg in the RE and 15 mm Hg in the LE without IOP-lowering medication. Central anterior chamber depth increased from 1.24 to 4.36 mm in the RE and from 2.27 to 4.35 mm in the LE. Uncorrected visual acuity was 20/20 in the RE and 20/33 in the LE, and best-corrected visual acuity was 20/20 in both eyes.

**Lessons:**

Emergency surgical peripheral iridectomy may serve as a rescue procedure in selected patients with medically uncontrolled acute angle closure when LPI is not feasible. Subsequent lens extraction may relieve residual lens-related anterior-segment crowding. However, this single case cannot establish the comparative effectiveness of the staged approach or whether peripheral iridectomy followed by lens extraction is the optimal treatment sequence.

## 1. Introduction

Acute angle closure is an ophthalmic emergency in which sudden iridotrabecular contact causes a rapid increase in intraocular pressure (IOP). Although the term acute angle-closure glaucoma is commonly used clinically, contemporary disease classification reserves glaucoma for eyes with glaucomatous optic neuropathy; an acute symptomatic episode without documented glaucomatous damage is more precisely termed acute primary angle closure (APAC) or an acute angle-closure crisis.^[1–3]^ Delayed IOP control may result in optic nerve injury, irreversible visual loss, corneal decompensation, and other ocular complications.^[1–3]^ Initial management aims to reduce IOP rapidly, control inflammation and symptoms, and permit definitive relief of pupillary block. Once the cornea is sufficiently clear, laser peripheral iridotomy (LPI) is generally the preferred definitive treatment, accompanied by evaluation and prophylactic management of the fellow eye.^[1–3]^

When medical therapy does not achieve adequate IOP control and severe corneal edema or marked iridocorneal apposition precludes LPI, alternative interventions must be individualized. Argon laser peripheral iridoplasty may be considered when corneal clarity permits laser delivery, whereas anterior chamber paracentesis can provide rapid but temporary pressure reduction. Surgical peripheral iridectomy can relieve pupillary block when laser treatment is not feasible. Primary lens extraction may also be considered in carefully selected patients, although surgery during the acute phase can be technically challenging.^[2–6]^ Alternatively, lens extraction may be deferred until the eye has stabilized, thereby reducing lens-related angle crowding and deepening the anterior chamber.^[7–9]^ Treatment selection should account for the mechanism of angle closure, corneal clarity, anterior chamber depth, lens status, extent of peripheral anterior synechiae, optic nerve status, surgical expertise, and anticipated procedural risk.

We describe a patient with APAC and medically uncontrolled IOP elevation in whom severe anterior-segment crowding and corneal edema precluded safe LPI. Emergency surgical peripheral iridectomy was performed first, followed by lens extraction after stabilization of the ocular condition. This case illustrates a possible staged management strategy for selected patients while recognizing the roles and limitations of alternative approaches.

## 2. Case presentation

A 61-year-old woman presented with a 7-hour history of right-eye pain, redness, and blurred vision accompanied by headache. At presentation, uncorrected visual acuity (UCVA) was 20/25 (0.10 logMAR) in both eyes (OU), improving to a best-corrected visual acuity (BCVA) of 20/22 (0.05 logMAR) in OU. IOP was 32 mm Hg in the right eye (RE) and 16 mm Hg in the left eye (LE). She had no history of a previous acute angle-closure episode, ocular trauma, or ocular surgery; was not taking any systemic or topical medication known to precipitate angle closure; and had no significant systemic comorbidity.

Examination of the RE revealed moderate-to-marked conjunctival injection, a mid-dilated pupil with a sluggish light response, mild diffuse epithelial and stromal corneal edema, and an extremely shallow peripheral anterior chamber, with near-apposition of the peripheral iris to the posterior corneal surface (Fig. 1A). Anterior chamber inflammation was graded as 1 + cells and 2 + flare. The optic disc was poorly visualized at presentation because of corneal edema. After the edema resolved, fundus examination showed an optic disc with sharp margins, normal pink coloration, and a vertical cup-to-disc ratio of approximately 0.4, without clinically apparent glaucomatous optic neuropathy. Static gonioscopy demonstrated iridotrabecular contact in all 4 quadrants (Shaffer grade 0). On indentation gonioscopy, the angle opened partially to Shaffer grades 1 to 2, depending on the quadrant, suggesting a substantial appositional component.

**Figure 1.**
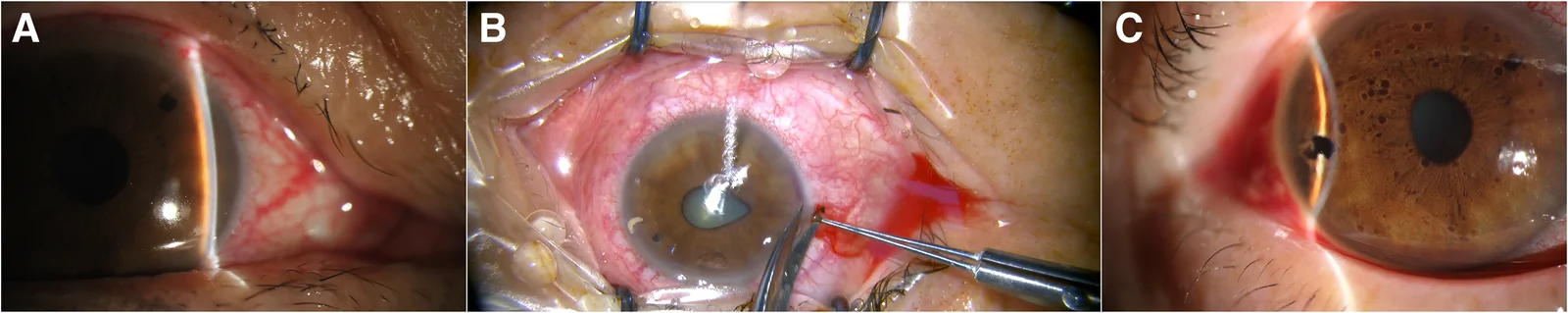
Clinical findings and emergency surgical peripheral iridectomy in the right eye. (A) Slit-lamp photograph at presentation showing conjunctival injection, corneal edema, and an extremely shallow peripheral anterior chamber, with near-apposition of the peripheral iris to the posterior corneal surface. (B) Intraoperative photograph showing the peripheral iris grasped and externalized through the scleral tunnel. (C) Postoperative slit-lamp photograph showing a patent, full-thickness peripheral iridectomy and deepening of the peripheral anterior chamber.

The asymptomatic LE had a shallow anterior chamber with anterior bowing of the peripheral iris. Static gonioscopy likewise demonstrated iridotrabecular contact in all 4 quadrants (Shaffer grade 0). On indentation gonioscopy, the angle opened to Shaffer grade 3 inferiorly, grade 2 superiorly and temporally, and grade 4 nasally. No glaucomatous optic neuropathy was evident.

Preoperative standard automated perimetry showed mild generalized sensitivity depression without a characteristic glaucomatous visual-field defect in either eye. Mean defect was 3.3 dB in the RE and 5.5 dB in the LE.

Manifest refraction was +0.75 to 1.25 × 70° in the RE and +2.75 to 1.75 × 90° in the LE. Axial length was 23.56 mm in the RE and 23.28 mm in the LE, while lens thickness was 5.61 and 5.20 mm, respectively. Central anterior chamber depth, measured from the corneal endothelium to the anterior lens surface, was 1.24 mm in the RE and 2.27 mm in the LE. These biometric findings indicated pronounced lens-related anterior-segment crowding, particularly in the affected eye.

The RE was diagnosed with APAC, whereas the LE was classified as a primary angle-closure suspect because of an occludable angle without elevated IOP, peripheral anterior synechiae, or glaucomatous optic neuropathy. Initial treatment of the RE comprised topical 1% pilocarpine every 30 minutes, 1% carteolol hydrochloride twice daily, and 1% brinzolamide 3 times daily.

Four hours after presentation, the patient’s symptoms worsened, and IOP in the RE increased to 58 mm Hg. She was subsequently treated with an intravenous infusion of 20% mannitol (250 mL; 50 grams) and topical 1% pilocarpine administered every 10 minutes for 1 hour and every 30 minutes thereafter. Systemic acetazolamide was not available for clinical use at the treating institution, and a topical α2-adrenergic agonist was unavailable in the emergency setting; consequently, neither medication was administered. As IOP remained markedly elevated, a second infusion of 20% mannitol at the same dose was administered 3.5 hours after the initial infusion. Persistent peripheral iridocorneal apposition, corneal edema, and an extremely shallow anterior chamber precluded the safe performance of LPI. Because symptoms persisted and IOP remained medically uncontrolled, emergency surgical peripheral iridectomy was performed approximately 15 hours after presentation.

### 2.1. Timeline

The clinical timeline and outcomes are summarized in Table 1.

**Table 1:** Clinical timeline and outcomes.

Time	Clinical status	Intervention	Outcome
7 h before presentation	Onset of right-eye pain, redness, blurred vision, and headache	None	Symptoms progressed
Presentation	IOP: 32/16 mm Hg (RE/LE); static gonioscopy: complete iridotrabecular contact in all quadrants of the RE; central ACD: 1.24/2.27 mm (RE/LE)	Topical 1% pilocarpine every 30 min, 1% carteolol twice daily, and 1% brinzolamide 3 times daily	Symptoms worsened; RE IOP increased
4 h after presentation	RE IOP: 58 mm Hg	First intravenous infusion of 20% mannitol (250 mL; 50 g); intensified topical pilocarpine	IOP remained markedly elevated
7.5 h after presentation	Persistent symptoms and markedly elevated IOP	Second intravenous infusion of 20% mannitol (250 mL; 50 g)	IOP remained markedly elevated, necessitating further intervention
Approximately 15 h after presentation	Corneal edema and an extremely shallow anterior chamber precluded safe LPI	Emergency surgical peripheral iridectomy in the RE	Pain and headache subsided; corneal edema resolved; IOP decreased to 11 mm Hg
Day 1 after RE iridectomy	Clear cornea; patent iridectomy; central ACD: 1.60 mm; IOP: 11 mm Hg	Topical 0.1% fluorometholone 4 times daily	RE stabilized
Day 3 after RE iridectomy	RE clinically stable	RE phacoemulsification with IOL implantation	No intraoperative complications
Day 6 after RE iridectomy	Occludable angle in the LE without glaucomatous optic neuropathy	LE phacoemulsification with IOL implantation and capsular tension ring insertion	No intraoperative complications
Approximately 6 weeks after bilateral lens extraction	IOP: 15 mm Hg (OU)	No IOP-lowering medication	UCVA: 20/25 (RE), 20/33 (LE); BCVA: 20/22 (RE), 20/20 (LE)
6 mo	IOP: 14/15 mm Hg (RE/LE)	No IOP-lowering medication	UCVA: 20/20 (RE), 20/33 (LE); BCVA: 20/20 (OU); central ACD: 4.36/4.35 mm (RE/LE); no adverse events

Note: Central ACD was measured from the corneal endothelium to the anterior lens surface preoperatively and to the anterior IOL surface postoperatively.

ACD = anterior chamber depth, BCVA = best-corrected visual acuity, IOL = intraocular lens, IOP = intraocular pressure, LE = left eye, LPI = laser peripheral iridotomy, OU = both eyes, RE = right eye, UCVA = uncorrected visual acuity.

### 2.2. Surgical technique

Under retrobulbar anesthesia, a 15° blade was advanced through the conjunctiva and sclera at the 9-o’clock position, approximately 1 mm posterior to the limbus, to create an oblique scleral tunnel. The blade was then carefully advanced into the anterior chamber, avoiding injury to the iris or crystalline lens. The anterior chamber was not actively maintained with irrigation, an ophthalmic viscosurgical device, or an anterior chamber maintainer. The incision was enlarged by approximately 1 mm with the same blade to permit instrument access. Fine-tipped iris forceps were introduced through the incision, and the peripheral iris was gently grasped and externalized (Fig. 1B). A full-thickness peripheral iridectomy approximately 0.5 mm in diameter was created at the corresponding site. The remaining iris tissue was carefully reposited, and the patency of the iridectomy was confirmed (Fig. 1C). A small volume of balanced salt solution was subsequently injected to deepen the anterior chamber. The wound was gently massaged, and no aqueous leakage was observed. At the conclusion of the procedure, digital palpation indicated physiologic globe tension.

Intraoperatively, aqueous humor flow through the iridectomy was observed, accompanied by immediate deepening of the peripheral anterior chamber. Postoperatively, the ocular pain and headache rapidly subsided, the corneal edema resolved, and IOP decreased to 11 mm Hg without further IOP-lowering medication. The iridectomy remained patent, and central anterior chamber depth (ACD) increased from 1.24 to 1.60 mm (Fig. 2A). Postoperatively, topical 0.1% fluorometholone was administered 4 times daily.

**Figure 2.**
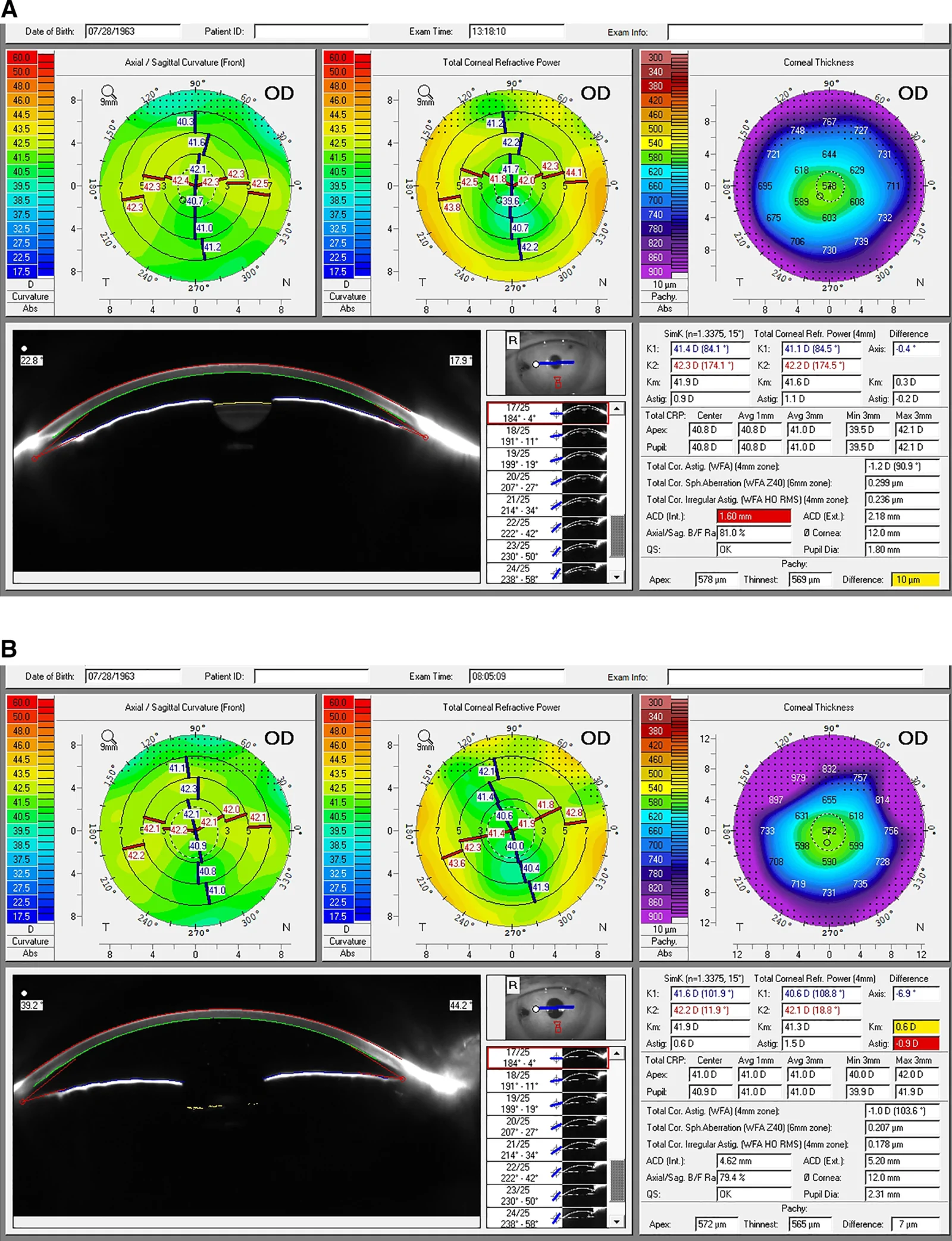
Scheimpflug anterior-segment images obtained with the Pentacam during staged treatment of the right eye. (A) After peripheral iridectomy but before lens extraction, the central anterior chamber depth was 1.60 mm. (B) At the first postoperative biometry assessment after phacoemulsification and intraocular lens implantation, the central anterior chamber depth was 4.62 mm. Before lens extraction, depth was measured from the corneal endothelium to the anterior lens surface; after lens extraction, it was measured from the corneal endothelium to the anterior intraocular lens surface.

After the ocular condition stabilized, we performed uncomplicated phacoemulsification with intraocular lens implantation in the RE 3 days after peripheral iridectomy. At the first postoperative biometry assessment, ACD was 4.62 mm (Fig. 2B). After right-eye phacoemulsification, a fixed-combination ophthalmic suspension containing 0.1% dexamethasone and 0.3% tobramycin was administered 4 times daily, tapered according to the postoperative inflammatory response, and discontinued within 1 month. On postoperative day 1, week 1, week 6, and month 6 after lens extraction, IOP was 13, 14, 15, and 14 mm Hg, respectively, without IOP-lowering medication. At the corresponding visits, UCVA was 20/25, 20/25, 20/25, and 20/20, respectively, and BCVA was 20/25, 20/20, 20/22, and 20/20, respectively.

Three days after right-eye lens extraction, the LE underwent phacoemulsification with intraocular lens implantation. After left-eye phacoemulsification, a fixed-combination ophthalmic suspension containing 0.1% dexamethasone and 0.3% tobramycin was administered 4 times daily, tapered according to the postoperative inflammatory response, and discontinued within 1 month. Lens extraction rather than prophylactic LPI or observation was selected because of the combination of complete iridotrabecular contact on static gonioscopy, marked lens-related anterior-segment crowding (lens thickness, 5.20 mm; ACD, 2.27 mm), and the anticipated visual and refractive benefits of lens removal after discussion of the available alternatives with the patient. Zonular weakness and associated capsular bag laxity were observed intraoperatively, prompting insertion of a capsular tension ring to stabilize and support the capsular bag. At 6 months, IOP was 15 mm Hg without IOP-lowering medication, UCVA was 20/33, and BCVA was 20/20. Central anterior chamber depth in the LE increased from 2.27 mm preoperatively (Fig. 3A) to 4.35 mm at 6 months after lens extraction (Fig. 3B). No procedure-related adverse events were documented in either eye during follow-up.

**Figure 3.**
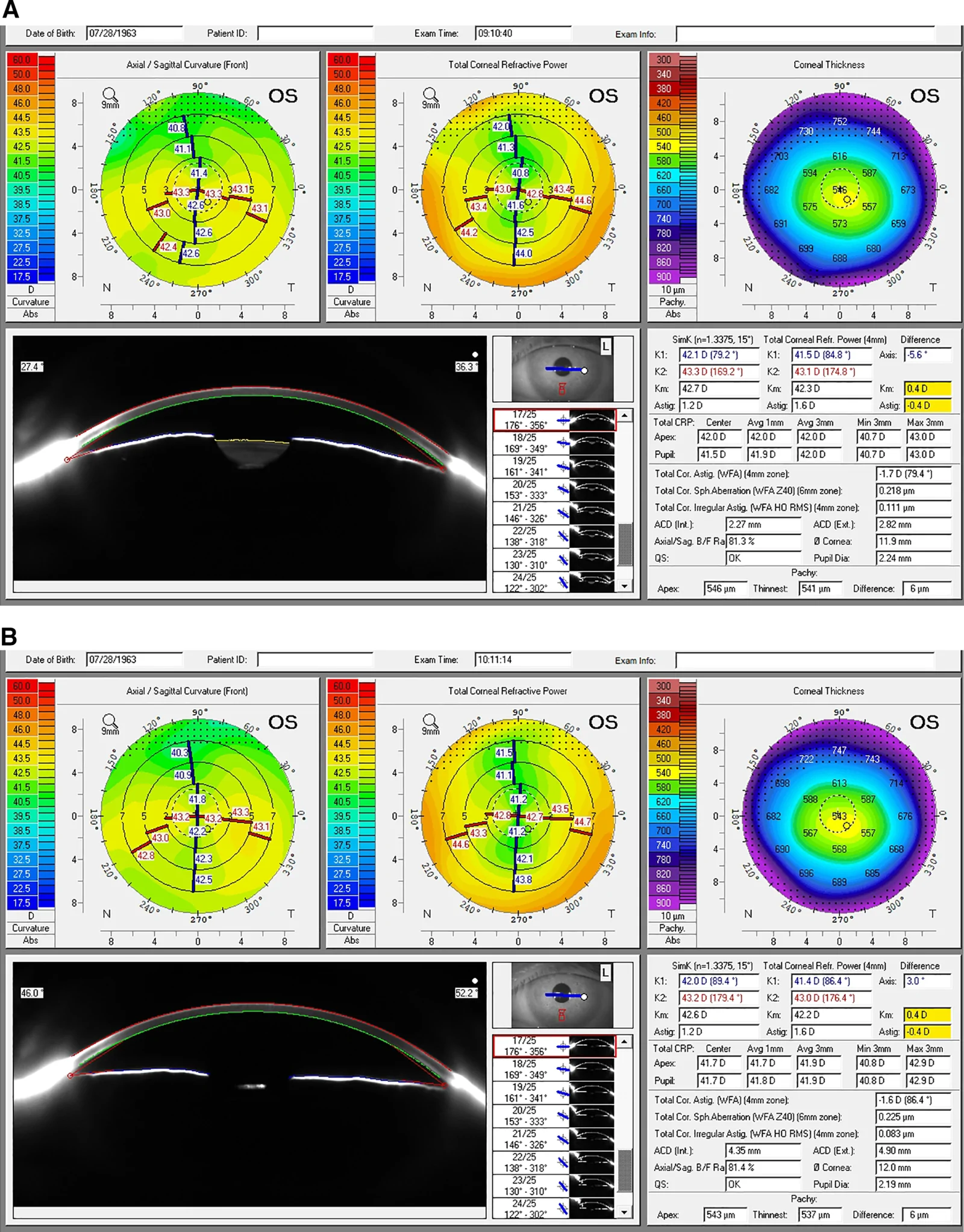
Scheimpflug anterior-segment images obtained with the Pentacam before and after lens extraction in the left eye. (A) Before lens extraction, the central anterior chamber depth was 2.27 mm. (B) At 6 mo after phacoemulsification and intraocular lens implantation, the central anterior chamber depth had increased to 4.35 mm. Before lens extraction, depth was measured from the corneal endothelium to the anterior lens surface; after lens extraction, it was measured from the corneal endothelium to the anterior intraocular lens surface.

Repeat standard automated perimetry approximately 2 months after surgery again showed no characteristic glaucomatous visual-field defect; mean defect was 2.9 dB in the RE and 4.5 dB in the LE. Because only 2 examinations were available and the baseline tests preceded cataract extraction, these small numerical differences were not interpreted as evidence of true functional change.

### 2.3. Ethics and consent

Formal ethics committee approval was not required because this single-patient case report was based exclusively on routine clinical care and involved no prospective research intervention or experimental procedure. Written informed consent was obtained from the patient for publication of the clinical information and accompanying images.

## 3. Discussion

This case describes an acute angle-closure crisis in which IOP increased during initial topical therapy and subsequently remained markedly elevated despite repeated intravenous mannitol, while corneal edema and an extremely shallow anterior chamber precluded safe LPI. Emergency surgical peripheral iridectomy was followed by rapid symptomatic relief, resolution of corneal edema, a reduction in IOP from 58 to 11 mm Hg, and deepening of the central anterior chamber from 1.24 to 1.60 mm. Subsequent lens extraction produced greater anterior chamber deepening, and IOP remained controlled without medication throughout 6 months of follow-up. These observations support the feasibility of a staged approach in carefully selected patients when immediate laser treatment is not possible and primary lens extraction during the acute attack is considered technically challenging and potentially higher risk.

The immediate priorities in an acute angle-closure crisis are to lower IOP, reduce corneal edema and inflammation, and relieve pupillary block before irreversible optic nerve or anterior-segment damage occurs.^[1–3,10]^ LPI is generally the preferred definitive treatment for pupillary-block–mediated angle closure once adequate corneal clarity has been achieved.^[1–3]^ In the present case, persistent corneal edema and peripheral iridocorneal apposition prevented adequate visualization for safe laser delivery. Argon laser peripheral iridoplasty can facilitate angle opening in some acute cases but similarly requires sufficient corneal clarity.^[4]^ Anterior chamber paracentesis can rapidly lower IOP, but its effect may be temporary because it does not eliminate the underlying pupillary-block mechanism.^[5]^ Entry into an extremely shallow anterior chamber also carries risks of iris, lens, and corneal endothelial injury. Intervention must therefore be individualized according to corneal clarity, anterior chamber depth, the presumed mechanism of closure, procedural availability, surgical expertise, and the urgency of IOP reduction.

Surgical peripheral iridectomy creates a direct pathway for aqueous humor flow from the posterior to the anterior chamber and can definitively relieve pupillary block when LPI is not feasible. In this patient, intraoperative aqueous flow through the iridectomy and immediate deepening of the peripheral anterior chamber were followed by marked IOP reduction and maintained iridectomy patency. This temporal association supports pupillary block as an important component of the acute episode. Although surgical peripheral iridectomy requires an appropriate operative setting and an anterior-segment surgeon, it may provide a practical rescue option when laser treatment cannot be performed. Available evidence remains limited, and further studies are needed to define the patients most likely to benefit from this approach.^[11]^

The biometric findings also indicated an important lens-related anatomical component. Before treatment, the affected eye had a lens thickness of 5.61 mm and a ACD of 1.24 mm. Peripheral iridectomy relieved pupillary block and increased anterior chamber depth to 1.60 mm but did not eliminate lens-related crowding. Lens extraction can deepen the anterior chamber, widen the angle, and reduce iridotrabecular contact by removing the volume and anterior position of the crystalline lens.^[7–10]^ Randomized evidence has shown advantages of early phacoemulsification over LPI alone after an APAC episode, and the EAGLE trial supports clear-lens extraction as an initial option in selected patients with primary angle closure or primary angle-closure glaucoma.^[7,8]^ The EAGLE trial, however, did not evaluate emergency lens extraction during an uncontrolled acute attack and should not be directly extrapolated to that setting. Phacoemulsification in an eye with severe corneal edema, markedly elevated IOP, a very shallow anterior chamber, and active inflammation can be technically difficult and may increase the risks of endothelial injury, iris trauma, capsular complications, and postoperative inflammation.

The staged strategy separated urgent relief of pupillary block from subsequent correction of lens-related crowding. Emergency peripheral iridectomy first reduced IOP and allowed the cornea and anterior segment to stabilize. Right-eye lens extraction was then performed 3 days later under improved operative conditions. At 6 months, ACD was 4.36 mm, and IOP was 14 mm Hg without medication. Although these anatomical and IOP outcomes are consistent with the expected effects of lens extraction, its independent contribution cannot be determined because peripheral iridectomy had already been performed and the acute inflammation had subsided before lens extraction.

The fellow eye had anterior bowing of the peripheral iris and iridotrabecular contact in all 4 quadrants on static gonioscopy, indicating a substantial risk of future angle closure despite the absence of symptoms, elevated IOP, peripheral anterior synechiae, or glaucomatous optic neuropathy. Management of the fellow eye is important because bilateral anatomical predisposition is common in primary angle-closure disease.^[1–3]^ In this patient, the choice of lens extraction over prophylactic LPI or observation reflected the combination of complete iridotrabecular contact, a 5.20-mm-thick lens, a ACD of 2.27 mm, and the anticipated visual and refractive benefits of lens removal after discussion of the alternatives. In this case, lens extraction in the fellow eye increased the ACD from 2.27 mm preoperatively to 4.35 mm at 6 months postoperatively, while IOP was 15 mm Hg without IOP-lowering medication. Nevertheless, lens extraction should not be regarded as mandatory for every primary angle-closure suspect. The choice among prophylactic LPI, lens extraction, close observation should account for lens status, angle anatomy, visual needs, age, comorbidities, and the individualized balance of benefits and risks.

The principal limitations of this report are its single-patient design, 6-month follow-up, and lack of serial gonioscopic or anterior-segment imaging assessment of angle anatomy. Larger studies with longer follow-up are needed to evaluate the durability and broader applicability of this staged approach.

## 4. Conclusion

Emergency surgical peripheral iridectomy may provide rapid IOP control in selected patients with medically uncontrolled acute angle closure when LPI is not feasible. Subsequent lens extraction may relieve residual lens-related anterior-segment crowding and support sustained IOP control. This case supports consideration of a staged approach tailored to the patient’s anatomy, clinical condition, and available surgical expertise.

## Author contributions

**Conceptualization:** Yu-Feng Yao.

**Data curation:** Yingjie Chen.

**Investigation:** Yingjie Chen.

**Methodology:** Yu-Feng Yao.

**Project administration:** Yu-Feng Yao, Xiaofeng Zhang.

**Writing – original draft:** Yuecong Yin.

**Writing – review & editing:** Yu-Feng Yao, Yuecong Yin.
